# Identification of the Phenicol Efflux Gene *fexB* and Its Co‐Occurrence With the Oxazolidinone/Phenicol Resistance Gene *optrA* in Avian *Campylobacter jejuni* Isolates From Tunisia

**DOI:** 10.1155/ijm/5554843

**Published:** 2026-03-02

**Authors:** Manel Gharbi, Chadlia Hamdi, Mohammed Abdo Saghir Abbas, Safa Hamrouni, Abderrazak Maaroufi

**Affiliations:** ^1^ Group of Bacteriology and Biotechnology Development, Laboratory of Epidemiology and Veterinary Microbiology, Institut Pasteur de Tunis, University of Tunis El Manar, Tunis, 1002, Tunisia, utm.rnu.tn; ^2^ Higher Institute of Biotechnology of Beja, University of Jendouba, Beja, 9000, Tunisia, uj.rnu.tn; ^3^ Unit of Vector Ecology, Pasteur Institute of Tunis, Tunis, 1002, Tunisia, pasteur.tn

**Keywords:** *Campylobacter jejuni*, *fexB*, multidrug resistance, *optrA*, Poultry

## Abstract

**Background:**

*Campylobacter jejuni*, a major foodborne and zoonotic pathogen linked to poultry, is showing rising resistance to last‐resort antimicrobials like oxazolidinones and phenicols. Such resistance threatens both animal and human health. Its ability to acquire genes from gram‐positive bacteria highlights the need for surveillance in avian reservoirs.

**Aim:**

This study aimed to characterize oxazolidinone‐ and phenicol‐resistance determinants among 197 *C. jejuni* isolates from avian sources in Tunisia.

**Methods:**

Isolates were studied by determining their antimicrobial susceptibility by disk diffusion method, and resistant isolates were screened by PCR for *fexA*, *fexB*, *floR*, RE‐*cmeABC*, *cfr*, and *optrA* genes.

**Results:**

Resistance rates were alarmingly high (57.6%–100%) across multiple antibiotic classes, and the majority were multidrug resistant. Among 155 (78.7%) chloramphenicol‐resistant isolates, the prevalence of *optrA*, *cfr*, *fexA*, *floR*, RE‐*cmeABC*, and *fexB* was 85%, 55%, 75%, 46%, 42%, and 22%, respectively, with most isolates harboring multiple genes. Phylogenetic analysis revealed close relatedness among Tunisian *optrA* and *fexB* sequences and similarities with those reported in *Enterococcus* spp., suggesting intergenus gene exchange.

**Conclusion:**

This study reports for the first time the detection of *fexB* gene and its co‐occurrence with *optrA* in *C. jejuni*, highlighting its potential role as a reservoir of transferable resistance genes. These findings underscore the urgent need for enhanced surveillance and antimicrobial stewardship within a One Health framework.

## 1. Introduction

*Campylobacter* *jejuni* is one of the leading bacterial causes of foodborne gastroenteritis worldwide, with poultry being recognized as the primary reservoir for human infection [1, 2]. Beyond its clinical importance as an enteric pathogen, *C. jejuni* has also emerged as a significant concern in the context of antimicrobial resistance (AMR) [3]. Over the past two decades, irrational and widespread use of antimicrobials in veterinary and agricultural settings has contributed to the alarming rise of multidrug‐resistant (MDR) *Campylobacter* strains [3, 4].

Among the antimicrobials of concern, phenicols (such as chloramphenicol [CHL] and florfenicol) and oxazolidinones (such as linezolid [LIN]) are considered critically important agents for both human and veterinary medicine. LIN, in particular, represents a last‐resort antibiotic in the treatment of severe and life‐threatening infections caused by MDR Gram‐positive pathogens, including *Staphylococcus aureus* and *Enterococcus* spp. [5, 6]. However, recent studies have reported the emergence of transferable LIN resistance genes, including *optrA*, *cfr*, and *poxtA*, in diverse bacterial species of human and animal origin [7–11]. The detection of such genes in foodborne zoonotic bacteria raises major One Health concerns, as they may act as hidden reservoirs capable of transmitting resistance across ecosystems [12].

In *Campylobacter*, several mechanisms of resistance to phenicols and oxazolidinones have been described, including efflux pumps, target‐site modifications, and, more recently, acquired resistance genes [13–17]. The plasmid‐mediated *optrA* gene, which confers resistance to both oxazolidinones and phenicols, has been increasingly identified in enterococci and staphylococci, but its occurrence in *Campylobacter* remains largely underexplored [14–18]. Globally, the *fexB* gene, a phenicol efflux gene initially described in enterococci, has only been reported in *C. coli* in our previous article (Tunisia) [16]; however, *fexA* gene has been commonly reported in *Campylobacter* spp isolates [13–15, 19].

Given the clinical and epidemiological importance of *Campylobacter*, the detection of transferable oxazolidinone/phenicol resistance genes in this genus represents a significant public health concern. Therefore, the present study aimed to investigate the prevalence of acquired oxazolidinone‐ and phenicol‐resistance determinants among poultry‐derived *C. jejuni* isolates from Tunisia. To our knowledge, this study reports for the first time the detection of the *fexB* gene in *C. jejuni* and its co‐occurrence with the *optrA* gene, underscoring the potential role of *Campylobacter* as a reservoir and vector of last‐resort AMR genes within the One Health framework.

## 2. Materials and Methods

### 2.1. Bacterial Isolates

A total of 197 *C. jejuni* isolates were recovered from 590 broiler chicken fecal samples and 143 environmental samples collected from 28 farms across three northeastern Tunisian governorates (Ariana, Ben Arous, and Nabeul), which together represent 29% of the national broiler production, between December 2016 and May 2018. These strains have been reported in our previous studies [20–24]. The farms, which included both intensive and semiextensive production systems, followed similar breeding and biosecurity protocols, with flock sizes ranging from 2000 to 18,000 birds per house. Samples consisted of cloacal swabs from chickens aged 15–40 days, as well as feces and water from the farm environment. All chickens were antibiotic‐free prior to sampling, and collections were performed using standard aseptic procedures, ensuring representative coverage of the Tunisian poultry sector.

### 2.2. Antimicrobial Susceptibility Testing

Antimicrobial susceptibility of all isolates was assessed using the disk diffusion method, following the recommendations of the European Committee on Antimicrobial Susceptibility Testing [25]. The panel of antimicrobial agents tested (Oxoid, Basingstoke, UK) included ampicillin (AMP, 10 μg), amoxicillin/clavulanic acid (AMC, 10/20 μg), gentamicin (GEN, 10 μg), streptomycin (SMN, 10 μg), kanamycin (KAN, 30 μg), nalidixic acid (NAL, 30 μg), ciprofloxacin (CIP, 5 μg), tetracycline (TET, 30 μg), erythromycin (ERY, 15 μg), azithromycin (AZM, 15 μg), CHL (30 μg), and LIN (10 μg). Isolates were considered MDR when they displayed resistance to at least three antimicrobial agents belonging to three or more distinct antibiotic classes, according to the criteria defined by Magiorakos et al. [26].

### 2.3. Extraction of Bacterial DNA

The genomic DNA of collected isolates was extracted using the boiling method [27]. Briefly, *Campylobacter* isolates were grown in 2 mL Bolton broth and plated on Karmali agar. *Campylobacter* colonies were then harvested and suspended in 100 μL·TE buffer (10 mM Tris, 1 mM EDTA, pH 8.0). Cell suspensions were heated at 100 °C for 10 min and then cooled at −20 °C for 5 min. Thereafter, cell suspensions were pelleted by centrifugation at 8000 rpm for 5 min. The supernatant containing DNA was collected, transferred into a new tube, and then stored at −20 °C until use for PCR experiments.

### 2.4. Screening of AMR Genes

CHL‐ and/or LIN‐resistant isolates were screened by PCR for resistance genes (*fexA, fexB, floR, RE-cmeABC, cfrA,* and *optrA*), while additional markers related to quinolones/fluoroquinolones, TETs, the *cmeABC* efflux pump, and macrolides were investigated in our previous studies (Supporting file Table S1) [20–24].

### 2.5. Sequencing and Phylogenetic Analysis of *optrA* and *fexB* Genes

Positive PCR products corresponding to the *optrA* and *fexB* genes were subjected to direct sequencing. Sequencing was carried out on an Applied Biosystems 3500 Genetic Analyzer using BigDye Terminator v3.1 chemistry. Raw sequence reads were assembled and manually edited with BioEdit Sequence Alignment Editor (Version 7.0.5.3), and the finalized sequences were compared to reference entries in the NCBI database using BLAST. The sequences obtained in this study have been deposited in GenBank under accession numbers PV656423 and PV656422 for *fexB* and PQ037488 and PQ037847 for *optrA*.

Phylogenetic analyses of the *fexB* (PV656423 and PV656422) and *optrA* (PQ037488 and PQ037847) genes were conducted to investigate their genetic relationships with previously reported sequences. Sequences generated in this study were aligned with homologous GenBank entries using ClustalW in MEGA11. Evolutionary histories were reconstructed using the maximum likelihood (ML) method with the Tamura 3‐parameter model, and initial trees were obtained via Neighbor‐Join and BioNJ algorithms applied to pairwise distance matrices. Tree robustness was evaluated with 1,000 bootstrap replicates. Sites containing gaps or missing data were excluded (complete deletion), yielding final datasets of 274 positions for *optrA* and 312 positions for *fexB*. The *fexB* tree was visualized and annotated in Interactive Tree of Life (iTOL v6), while the *optrA* tree was generated and displayed directly in MEGA11.

Antimicrobial susceptibility data obtained from disk diffusion testing were processed using descriptive statistical methods. For each antibiotic, resistance rates were calculated as the proportion of resistant isolates among the total sample (*n* = 197). These data were visualized in a heatmap to illustrate the relative prevalence of resistance across antimicrobial classes. To investigate coresistance, recurrent combinations of resistance to two or more antibiotics were identified, and the most frequent multidrug resistance profiles were represented in a second heatmap. This visualization enabled the detection of resistance clusters and associations between different antimicrobial classes. Both heatmaps were generated using the Seaborn package in Python (v3.10), with color gradients scaled according to resistance rates or coresistance frequencies. This analytical approach provided a comprehensive overview of resistance patterns and facilitated the interpretation of multidrug resistance dynamics in *C. jejuni* isolates.

### 2.6. Data Analysis

Antimicrobial susceptibility data from disk diffusion tests were analyzed using descriptive statistics, with resistance rates calculated for each antibiotic as the proportion of resistant isolates (*n* = 197). Heatmaps were used to visualize resistance prevalence across antimicrobial classes and to display the most frequent multidrug resistance profiles, highlighting coresistance patterns and associations among antibiotics. Both heatmaps were generated with Seaborn in Python (v3.10), using color gradients scaled to resistance rates or coresistance frequencies. This approach provided a clear overview of resistance patterns and facilitated interpretation of multidrug resistance dynamics in *C. jejuni* isolates.

## 3. Results

### 3.1. AMR Rates, MDR Phenotypes, and Occurrence of Resistance Genes

**FIGURE 1:**
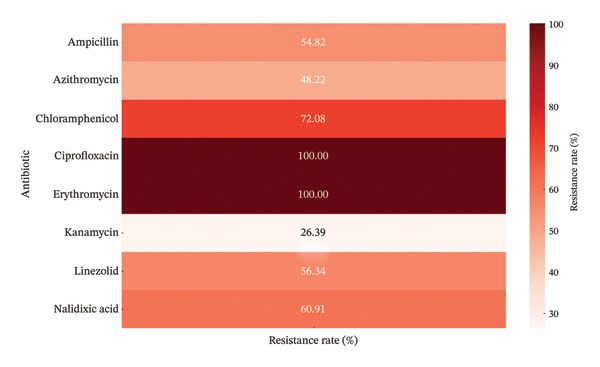
Resistance rates of *C. jejuni* isolates (*n* = 197) to tested antibiotics.

**FIGURE 2:**
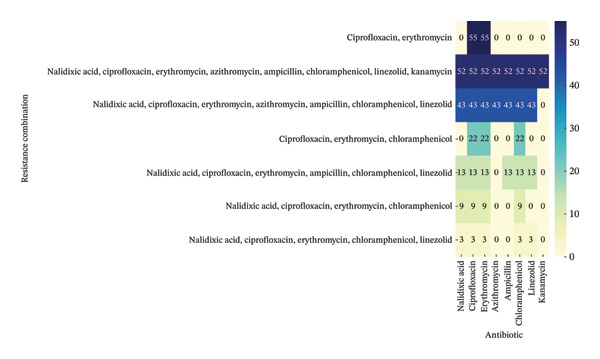
Number of isolates exhibiting co‐resistance profiles among multidrug‐resistant *C. jejuni* isolates.

The analysis of AMR profiles in the 197 *C. jejuni* isolates revealed alarmingly high resistance rates to several key antibiotics (Figure 1). All isolates were resistant to CIP and ERY (*n* = 197, 100%), while substantial resistance levels were also observed for CHL (*n* = 142, 72.08%), NAL acid (*n* = 120, 60.91%), LIN (*n* = 111, 56.34%), and AMP (*n* = 108, 54.82%). Moderate resistance was noted for AZM (*n* = 95, 48.22%) and KAN (*n* = 52, 26.39%). Investigation of coresistance patterns (Figure 2) confirmed these trends, with the most frequent combination involving CIP and ERY (*n* = 55, 27.9%). Moreover, severe multidrug resistance profiles, involving up to seven or eight antibiotics (fluoroquinolones, macrolides, β‐lactams, phenicols, oxazolidinones, and aminoglycosides), were identified in 43 (44.3%) to 52 (26.39%) isolates, respectively.

**FIGURE 3:**
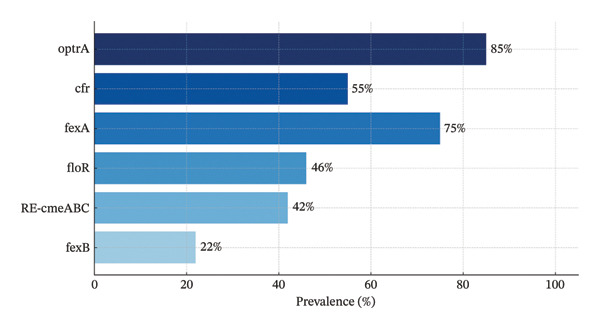
Prevalence of resistance genes among the chloramphenicol‐resistant *C. jejuni* isolates (*n* = 155).

Among the 155 *C. jejuni* isolates resistant to CHL, molecular screening revealed a high prevalence of resistance genes with notable variability across markers (Figure 3). The *optrA* gene was the most frequently detected (*n* = 167, 85%), followed by *fexA* (*n* = 148, 75%) and *cfr* (*n* = 108, 55%), confirming the widespread dissemination of oxazolidinone‐ and phenicol‐associated resistance determinants. Intermediate frequencies were observed for *floR* (*n* = 91, 46%) and *RE*‐*cmeABC* (*n* = 83, 42%), while *fexB* was less commonly detected (*n* = 44, 22%). Importantly, the majority of isolates (≥ 80%) coharbored multiple genes.

### 3.2. Sequencing and Phylogenetic Analysis of *fexB* and *optrA* Genes

**FIGURE 4:**
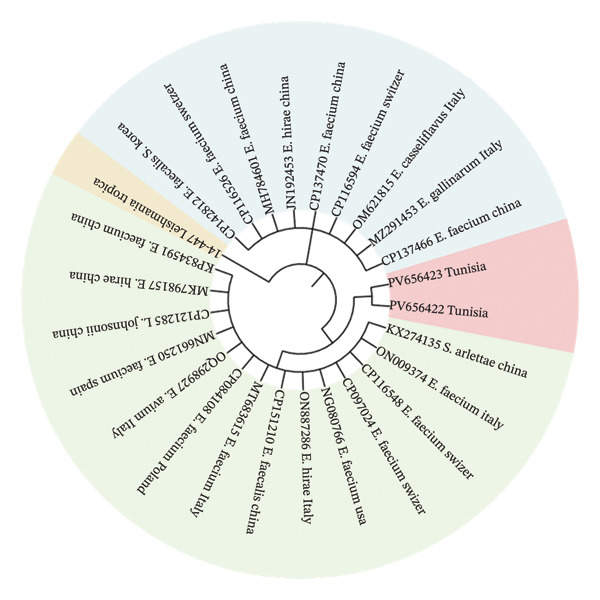
Evolutionary analysis of the *fexB* gene by maximum likelihood. The tree with the highest log likelihood (−1343.98) was inferred using the tamura 3‐parameter model. Initial trees were generated automatically via neighbor‐join and BioNJ algorithms on pairwise distance matrices, and the topology with the superior likelihood was selected. The model allowed for some sites to be invariable ([+I], 25.55%). Percentages next to branches indicate the frequency of clustering across replicate trees, and numbers at internal nodes show the proportion of sites with at least one unambiguous base in descendant clades. Twenty‐six nucleotide sequences (454 positions) were analyzed using MEGA11.

The phylogenetic tree based on *fexB* sequences revealed that the two Tunisian’s *fexB* sequences (sequences called *C. jejuni* PV656423 and *C. jejuni* PV656422) clustered together in a distinct subclade, demonstrating a high degree of similarity between the two sequences (Figure 4). These *fexB* sequences grouped in close proximity to *fexB* sequences from *E. faecium* (Italy and China) and *E. gallinarum* (Italy) strains.

**FIGURE 5:**
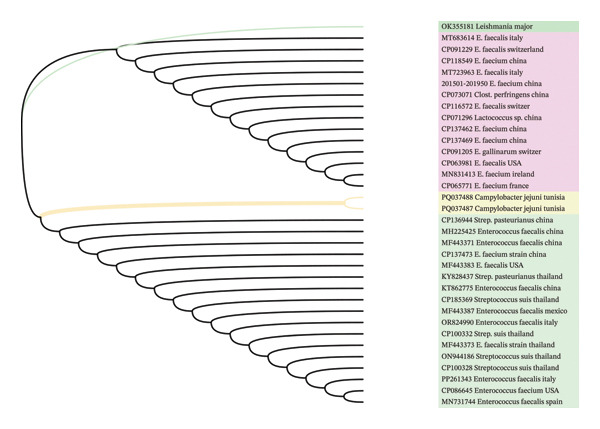
Phylogenetic analysis of the *optrA* gene. The evolutionary history was inferred using the maximum likelihood method with the Tamura 3‐parameter model. The tree with the highest log likelihood (−972.17) is shown. Initial trees were generated using neighbor‐Join and BioNJ algorithms, and the best topology was selected. The model allowed for invariant sites ([+I], 0.00%). The analysis included 34 nucleotide sequences, with all positions containing gaps or missing data removed, resulting in a final dataset of 274 positions. Analyses were performed in MEGA11.

The phylogenetic tree of *optrA* sequences revealed that the two Tunisian’s *optrA* sequences (PQ037488 and PQ037847) clustered together within a distinct subclade, reflecting their high genetic similarity (Figure 5). These sequences formed a clearly separated branch from other *optrA* alleles included in the dataset, indicating a unique genetic signature of the Tunisian isolates. Interestingly, the Tunisian’s *optrA* sequences were phylogenetically close to *optrA* genes reported in *Enterococcus faecalis* and *E. faecium* isolates from China, Italy, and the United States. In contrast, they were more distantly related to *optrA* alleles identified in streptococci (*Streptococcus pasteurianus*, *S. suis*) and other bacterial genera, suggesting evolutionary divergence and multispecies dissemination of this resistance determinant.

Overall, the topology indicates that the emergence of *optrA* in Tunisian *C. jejuni* isolates is not an isolated event but part of a wider international dissemination of this gene. The detection of *optrA* in *C. jejuni* is of particular concern, as it confers resistance to oxazolidinones (LIN) and phenicols, thus compromising last‐resort treatment options. These findings highlight the importance of continuous genomic surveillance under a One Health framework, given the risk of interspecies and interenvironmental gene transfer.

## 4. Discussion

Poultry production systems are significant interfaces between humans, animals, and the environment, making them major reservoirs for antimicrobial‐resistant bacteria [28]. Intensive farming practices, together with the extensive use of antibiotics for growth promotion and disease prevention, provide strong selective pressures that promote the emergence and spread of resistant pathogens [29, 30]. AMR investigation in avian settings is therefore crucial to understand the mechanisms underlying multidrug resistance, identify possible threats to human health, and develop effective One Health strategies.

According to the reported results in this study, the high prevalence of AMR observed among poultry‐derived *C. jejuni* isolates underscores a critical public health concern. The widespread resistance to CIP and ERY, together with high levels of resistance to CHL, NAL acid, LIN, and ampicillin, implies significant selective pressure within chicken production systems and a gradual loss of therapeutic efficacy. In addition, several MDR phenotypes were observed among the isolates. Globally, comparable patterns of MDR in avian *C. jejuni* have been reported in several countries, reflecting a global trend driven by the intensive use of antimicrobials in food animals [31, 32]. MDR phenotypes mainly include resistance towards fluoroquinolones, macrolides, and β‐lactams, highlighting the repeated emergence of strains capable of surviving multiple classes of antibiotics. The presence of MDR *C. jejuni* in poultry not only jeopardizes the efficacy of current therapeutic options for human campylobacteriosis but also suggests that poultry production systems act as strong reservoirs for the maintenance and spread of resistance determinants.

The molecular screening of CHL‐resistant *C. jejuni* isolates revealed an alarming complex resistome, emphasizing the diverse nature of AMR in poultry‐associated strains. The significant incidence of *optrA* (85%), *fexA* (75%), and *cfr* (55%) in the 155 resistant isolates indicates that oxazolidinone‐ and phenicol‐associated resistance determinants are broadly distributed across these populations. The high frequency of *optrA* gene is significant since it encodes resistance to oxazolidinones (e.g., LIN) and phenicols (e.g., CHL, florfenicol), suggesting the likelihood of reduced susceptibility to last‐resort antibiotics such as LIN. The frequent co‐occurrence of *optrA*, *fexA*, and *cfr* suggests that these genes may be physically linked on mobile genetic elements, such as plasmids or transposons, which facilitates horizontal gene transfer (HGT) within *C. jejuni* populations and potentially across bacterial species [33, 34]. The intermediate frequencies of *floR* (46%) and *RE-cmeABC* (42%) genes demonstrate the variability of CHL resistance mechanisms in the studied population. *floR* gene encodes an efflux pump that mediates resistance to florfenicol and CHL [35], and the RE‐cmeABC efflux system has been associated with broad‐spectrum resistance, including phenicols, macrolides, and TETs [36]. The relatively low detection rate of *fexB* (22%) gene indicates that, while less common, it contributes to the overall multidrug resistance background, particularly when co‐occurring with other determinants. The finding that the majority of isolates (≥ 80%) coharbored several resistance genes highlights the additive or synergistic impact of these determinants. This may increase bacterial survival under antimicrobial pressure and allow the dissemination of highly resistant *C. jejuni* clones. The aforementioned results have significant ramifications. First, the presence of *optrA*, *fexA*, and *cfr* genes in poultry‐derived *C. jejuni* suggests that resistance mechanisms previously described in gram‐positive bacteria, such as *Enterococcus* spp. and *Staphylococcus* spp., are now present in gram‐negative zoonotic pathogens, most likely *via* plasmids and other mobile genetic elements [6, 7, 37, 38]. Second, the presence of multiple resistance genes in a single isolate increases the risk of treatment failure in both human and veterinary medicine. Collectively, the prevalence and co‐occurrence of these resistance genes in CHL‐resistant *C. jejuni* isolates underscore a dynamic resistome shaped by intensive antimicrobial use, HGT, and selective pressure in avian environments, emphasizing the urgency of a One Health approach to combat the emergence and spread of multidrug‐resistant *Campylobacter*.

Phylogenetic analysis of the sequences of *fexB* and *optrA* further confirmed the enterococcal origin of these genes. Indeed, the clustering of Tunisian *C. jejuni fexB* sequences (PV656423 and PV656422) inside a discrete subclade demonstrates substantial sequence conservation, implying that these isolates may have had a recent common ancestor or been under similar selective pressures in the poultry environment. The close phylogenetic proximity of these sequences to *fexB* genes from *E. faecium* and *E. gallinarum* from Italy and China raises the possibility of interspecies HGT, highlighting again the ability of resistance determinants to move between gram‐positive and gram‐negative bacteria. This finding is especially interesting because *fexB* is generally linked with enterococci [9, 39], indicating that *C. jejuni* may have acquired such genes from enterococcal reservoirs within the avian microbiota, presumably via plasmids, transposons, or integrative conjugative elements [40].

Similarly, the phylogenetic analysis of *optrA* sequences revealed that the two Tunisian *C. jejuni* isolates (PQ037488 and PQ037847) were tightly grouped inside a separate subclade, indicating close genetic relatedness and a common evolutionary origin or acquisition event. The formation of a unique branch from other *optrA* alleles suggests that these Tunisian *optrA* sequences may represent a novel local variant or a region‐specific modification of the gene, most likely influenced by selective pressures in the chicken production setting. Such genetic difference could be the result of recombination or point mutations following the horizontal acquisition of *optrA*, indicating continuous microevolution in response to antibiotic pressure. The Tunisian *optrA* alleles are closely related to those found in *E. faecalis* and *E. faecium* isolates from China, Italy, and the United States, reinforcing the hypothesis of interspecies transmission via mobile genetic elements. The *optrA* gene has been predominantly identified in *Enterococcus* spp. but is increasingly detected in gram‐negative bacteria, including *Campylobacter* [14–18]. This pattern suggests that *Enterococcus* species may act as genetic reservoirs for *optrA*, facilitating its mobilization into other taxa through conjugative plasmids, transposons (such as Tn*6674* or Tn*554*‐like elements), or integrative conjugative elements [9, 10, 41]. Conversely, the distant relationship between Tunisian *optrA* alleles and those found in streptococci (*S. suis, S. pasteurianus*) and other bacterial *taxa* suggests some evolutionary divergence and lineage‐specific adaptation. This finding lends acceptance to the idea of *optr’s* multispecies dissemination, in which the gene spreads across phylogenetically divergent bacterial hosts yet evolves independently after being integrated into different genomic or plasmid backgrounds [9, 10, 41, 42]. Such diversity underscores the dynamic nature of resistance gene ecology, which is driven by HGT events within complex microbiomes like those found in chicken.

## 5. Conclusion

This study reveals a worrying prevalence of multidrug resistance among poultry‐derived *C. jejuni* isolates in Tunisia, notably against fluoroquinolones, macrolides, and oxazolidinones. The co‐occurrence of transferable determinants such as *optrA* and *fexA*, alongside the first identification of *fexB* in *C. jejuni*, highlights its emerging role as a reservoir of critical resistance genes. The phylogenetic relatedness of Tunisian *optrA* and *fexB* alleles with enterococcal counterparts provides compelling evidence of interspecies gene transfer. Collectively, these findings underscore the urgent need for integrated One Health surveillance and robust antimicrobial stewardship within the poultry production system.

## Author Contributions

Manel Gharbi: conceptualization, methodology, data curation, validation, formal analysis, and writing original draft and editing. Mohammed Abdo Saghir Abbas, Chadlia Hamdi, and Safa Hamrouni: data curation and visualization. Abderrazak Maaroufi: conceptualization, methodology, supervision, project administration, and writing–review and editing.

## Funding

This work was financed by funding provided to the Laboratory of Epidemiology and Veterinary Microbiology (LR16IPT03) by the Tunisian Ministry of Higher Education and Scientific Research.

## Disclosure

All authors have read and agreed to the published version of the manuscript.

## Ethics Statement

The study was approved by the Biomedical Ethics Committee of the Pasteur Institute of Tunis, with reference number: 2018/12/I/LR16IPT.

## Conflicts of Interest

The authors declare no conflicts of interest.

## Supporting Information

Supporting Table S1 provides detailed information on the primers used for the PCR amplification of antimicrobial resistance genes (*floR*, *fexA*, *fexB*, *cfr*, *optrA*, and *cmeABC*). The table includes the primer sequences, amplification conditions, and the expected product sizes.

## Supporting information


**Supporting Information** Additional supporting information can be found online in the Supporting Information section.

## Data Availability

The data that support the findings of this study are available on request from the corresponding author. The data are not publicly available due to privacy or ethical restrictions.
