# Supplementary material for: Identification of the Phenicol Efflux Gene fexB and Its Co‐Occurrence With the Oxazolidinone/Phenicol Resistance Gene optrA in Avian Campylobacter jejuni Isolates From Tunisia
Source: Int J Microbiol. 2026 Mar 2;2026:5554843. doi: 10.1155/ijm/5554843 (PMC12954346; doi:10.1155/ijm/5554843)
Supplement: Supplementary file 1 — Supporting Information Additional supporting information can be found online in the Supporting Information section. [file IJM-2026-5554843-s001.doc]

**Supplementary file**

**Table S1.** Primers used for PCR reactions [20-24].

| **Gene** | **Primers (5’ - 3’)** | **Conditions of amplification** | **Size (bp)** | |
| --- | --- | --- | --- | --- |
| *floR* | F- CGCCGTCATTCCTCACCTTC  R- GATCACGGGCCACGCTGTGTC | 94°C/5min; 35 cycles : 94°C/30s,50°C/30s,72°C/1min ; 72°C/5min | | 215 |
| *fexA* | F- TTGGGAAGAATGGTTCAGGG  R- ATCGGCTCAGTAGCATCACG | 95°C/5min; 30 cycles : 95°C/30s,50°C/30s,72°C/30s ; 72°C/5min | | 977 |
| *fexB* | F- ACTGGACAGGCAGGCTTAAT  R- CCTGCCCCAAGATACATTGC | 95°C/5min; 30 cycles : 95°C/30s,57°C/30s,72°C/30s ; 72°C/5min | | 319 |
| *cfr* | F- GGGAGGATTTAATAAATAATTTTGGAGAAACAG  R- CTTATATGTTCATCGAGTATATTCATTACCTCATC | 93°C/5min ; 35 cycles : 93°C/1min,58°C/1min,72°C/1min ; 72°C/5min | | 580 |
| *optrA* | optrA-F- AGGTGGTCAGCGAACTAA  optrA-R- ATCAACTGTTCCCATTCA | 95°C/3min; 30 cycles: 94°C/30s,55°C/30 s,72°C/90s; 72°C/5min | | 1395 |
| RE-*cmeABC* | F-CGTATTGCACGATTATTTGGAC  R-ATCGTTATCAAACCCTCTATGTGCC | 94°C/5min ; 35 cycles : 94°C/1min,54°C/1min,72°C/1min ; 72°C/5min | | 742 |
